# Motivational factors for blood donation, potential barriers, and knowledge about blood donation in first-time and repeat blood donors

**DOI:** 10.1186/s12878-018-0130-3

**Published:** 2018-12-20

**Authors:** Shamsudeen Mohammed, Harry Barton Essel

**Affiliations:** 1 Department of Nursing, College of Nursing and Midwifery, Post office box 10, Nalerigu, Ghana; 2 Educational Innovations in Science and Technology, Kwame Nkrumah University of Science and Technology, Kumasi, Ashanti Region Ghana

**Keywords:** Blood donors, Blood donation, Motivators, Barriers, Knowledge, Repeat donors, First-time donors, Tamale teaching hospital, Ghana

## Abstract

**Background:**

Blood transfusion is an essential component of the health care system of every country and patients who require blood transfusion service as part of the clinical management of their condition have the right to expect that sufficient and safe blood will be available to meet their needs. However, this is not always the case, especially in developing countries. To recruit and retain adequate regular voluntary non-remunerated blood donors the motivators and barriers of donors must be understood. Equally important to this goal is the knowledge of blood donors.

**Methodology:**

A cross-sectional study was conducted at the donor clinic of Tamale Teaching Hospital in the Northern Region of Ghana from 06 January to 02 February 2018. Purposive sampling technique was used to sample 355 eligible first-time and repeat whole blood donors. Data were collected face-to-face with a 27-item self-administered questionnaire. Chi-square test was used to determine the association between donor status and the motivators of blood donation, barriers to blood donation and the socio-demographic characteristics of donors.

**Results:**

Out of the 350 donors, 192(54.9%) were first-time blood donors while 158 (45.1%) were repeat donors. Nearly all the donors, 316(90.3%), indicated they were motivated to donate when someone they know is in need of blood. Over four-fifths of the donors endorsed good attitude of staff (*n* = 291, 83.4%) and the desire to help other people in need of blood (*n* = 298, 85.1%) as motivators. Approximately two-thirds, 223(63.7%), of the donors endorsed poor attitude of staff as a deterrent to blood donation. More than half of the donors considered the level of privacy provided during pre-donation screening (*n* = 191, 54.6%) and the concern that donated blood may be sold 178(50.9%) as deterrents. Only a little over one-third of the donors knew the minimum age for blood donation (n = 126, 36.0%) and the maximum number of donations per year (n = 132, 37.7%).

**Conclusion:**

Our findings suggest that public education on blood donation, regular prompts of donors to donate when there is a shortage, and friendly attitude of staff have the potential to motivate donors and eliminate barriers to blood donation.

## Background

Blood transfusion is an essential component of the healthcare system of every country and patients who require blood transfusion service as part of the clinical management of their condition have the right to expect that sufficient and safe blood will be available to meet their needs. However, this is not always the case, especially in developing countries. In many developing countries, there is a widespread shortfall between blood requirements and blood supplies and as a result, many patients die or suffer unnecessarily because they do not have access to blood and blood products [1]. The most affected are women and children. Blood transfusion in developing countries is often used to manage children with severe anaemia and women with pregnancy-related bleeding [2]. In 2017 about 65% of blood transfusions in low-income countries were for children under the age of 5 years [3]. In Ghana, more than 75% of donor blood in rural areas and 50% in urban areas are transfused to children under 5 years and women of childbearing age [4].

The high maternal and child mortality rates attributed to pregnancy related-complications, severe malaria, and anaemia, are evidence of the magnitude of the unmet need for blood transfusion in Africa [2]. About 34% of maternal deaths in Africa are still attributed to severe bleeding during and after childbirth [5]. Furthermore, man-made natural disasters, road traffic accidents, and armed conflicts further increase the demand for blood transfusion in Africa. Regrettably, many countries in the region collect less than half of the blood needed to meet the transfusion requirements of their populations and predictably, access to blood remains a challenge in many African countries including Ghana [2, 6]. Of the estimated 250, 000 units of blood required annually to meet the blood transfusion demand of patients in Ghana, 160, 624 units were collected in 2016. The majority of the donors were family replacement donors. Only 36.2% of the donors were voluntary non-remunerated blood donors [7, 8].

According to the World Health Organisation (WHO), voluntary non-remunerated blood donation is the surest approach to ensure sufficient supply of safe blood to meet national requirement of blood transfusion. However, only 62 countries have blood supplies based on close to 100% voluntary non-remunerated blood donations [9]. Blood donation systems in most countries largely depend on family replacement donors who donate blood to help a friend or family member in need of blood transfusion. These systems are rarely able to meet clinical demands for blood [1]. Nonetheless, family and/or replacement donors continue to dominate in sub-Saharan Africa generally because some society’s belief blood is a precious sacred substance common to family and should be preserved in the family and not donated to strangers. As a result, blood is only donated to save the life and relieve the suffering of a hospitalised relative. Additionally, people who believe behavioural traits can be transferred through blood prefer to receive blood from a known person or family member. Moreover, replacement donation is less costly compared with blood from a volunteer donor [10–12].

For a hospital to recruit and retain adequate regular voluntary non-remunerated blood donors the motivators and deterrents of the donors must be understood. However, a literature search revealed inadequate studies in the area, particularly in Ghana. Out of 35 studies reviewed by Asamoah-akuok et al. [11], only three focused on motivators and deterrents of blood donors. Equally important to the goal of recruiting adequate voluntary donors is the knowledge of blood donors. Information about blood donation process demystifies myths and fears related to blood donation. People are motivated to donate when they are well informed about the process and the life-saving benefits of given blood [1]. Asamoah-akuok et al. found that the main deterrent to blood donation in sub-Saharan Africa is fear due to lack of knowledge and discouraging cultural and religious beliefs about blood donation [11]. It is against this backdrop that this study assessed first-time and repeat blood donors’ motivators, barriers to donation, and knowledge about blood donation at the Tamale Teaching Hospital to understand the factors that motivate or discourage blood donation and to suggest cogent interventions to increase recruitment and retention of an adequate number of voluntary non-remunerated donors.

## Methods

### Study design, study site, and study population

A cross-sectional study was conducted at the blood donation centre (donor clinic) of Tamale Teaching Hospital (TTH) in the Northern Region of Ghana. The hospital serves patients largely from the Upper East, Upper West, and Northern Regions of Ghana. It is the only tertiary health facility in the three regions and serves as the teaching hospital for the medical school of University for Development Studies, Tamale campus. The hospital function in three important areas 1) medical education and training of physicians and other health professionals 2) provision of high-quality clinical care including specialised services and 3) undertake research into health issues for improving health care. All persons who donated blood at the donor clinic of the hospital from 06 January to 02 February 2018 constituted the population for the study. Blood donors were enlisted if they were above 18 years, irrespective of gender, and willing to participate in the survey. Non-donors at the clinic and donors who were unwilling to give consent were excluded.

### Sampling technique and sample size

Purposive sampling technique was used to recruit three hundred and fifty eligible first-time (persons donating for the first time) and repeat (donors who have donated two or more times) whole blood donors at the donor clinic of Tamale Teaching Hospital. Single population proportion formula was used to determine the sample size of 355 based on 36% prevalence of knowledge about blood donation [13], 95% confidence interval, and 5% degree of error. Donors were assessed for eligibility, informed about the purpose of the study, and invited to participate in the study after a routine pre-donation screening. The study protocol was reviewed and approved by the institutional research and development committee of Tamale Teaching Hospital. Participation was voluntary and participants indicated consent by signing a consent form after adequate information was provided on the purpose of the study, its possible benefits, and their role in the study. Additionally, participants were informed they could withdraw from the study at any time. Sufficient time was allowed for participants to decide whether to participate in the study. We ensured confidentiality of the study data and maintained the anonymity of the study participants.

### Data collection tool

Data were collected face-to-face with a 27-item self-administered questionnaire. It was designed after a comprehensive literature review to include relevant variables from previously published studies [11, 14–18]. The questionnaire assessed the following four categories: Socio-demographic characteristics of participants (7 items), motivators of blood donation (6 items), barriers to blood donation (9 items), and knowledge about blood donation (5 items). Table 1 presents the variables that were collected in each category. The knowledge questions were constructed based on information from the National Blood Donation service of Ghana. Two professionals in the area of blood donation reviewed the questionnaire and deemed it content valid. Recommended amendments after the review were made to improve the instrument. We pretested the questionnaire on 15 outpatient to ascertain the clarity and practicability of the questionnaire and to identify poorly constructed items. Relevant changes were made after the pre-test. Two final year nursing students, who were trained on how to administer and record responses, administered the questionnaire to the donors. The principal investigator supervised the data collection.

**Table 1:** Variables collected in each category of the questionnaire

Category	Variables collected
1. Participants socio-demographic characteristics	Age, sex, marital status, religion, education, employment, donor status
2. Motivators of blood donation	when someone I know is in need, good attitude staff, incentives, appeals on radio, television, or from a famous person, a reminder to donate when there is a shortage, and to help a person in need
3. Potential barriers to blood donation	Poor attitude of staff, level of privacy during screening, fear of weakness, fear of needles/pain, fear of contagion, fear of feeling faint or dizzy, the inconvenience of donors’ clinic, donated blood may be sold, and absence of incentives
4. Knowledge of blood donation	Minimum age for blood donation, maximum number of donations in a year, interval between two blood donations, does donated blood expire, and is a person positive for HIV/AIDS eligible to donate blood

### Data management and analysis

Collected data were checked for completeness, cleaned, coded, and entered into Microsoft Excel spreadsheet before exported into Stata v14 for analysis. Five [5] questionnaires were disqualified for incompleteness. We analysed 350 completed questionnaires. For descriptive statistics, frequency, percentage, mean, and standard deviation (SD) were computed. Using donor status (first- time and repeat) as a categorical dependent variable, the association of donor status and socio-demographic characteristics, motivators of blood donation, and barriers to blood donation was determined using Pearson’s chi-square. For independent variables that were small (expected values less than 5) Fisher’s exact test was used to determine the association. For the Pearson’s chi-square and Fisher’s exact tests, proportions, percentages, chi-values, and *P* values were presented in tables. The significance level was set at 0.05.

## Results

### Background characteristics

Out of the 355 questionnaires that were administered, five [5] questionnaires were disqualified for incompleteness. We analysed a total of 350 completed questionnaires. Participants’ background characteristics are presented in Table 2. Out of the 350 donors, 192(54.9%) were first-time blood donors. The remaining 158 (45.1%) were repeat donors. The majority, 246 (70.3%), of the donors were in the age range of 20–35 years. Only 11(3.1%) donors were older than 50 years. Mean age of donors in this study was 29.2(SD = 9.1) years. Most, 246 (73.1%), of the donors were males. Only 94(26.9%) were females. Among the participants, 142(40.6%) were never married while 182 (52.0%) were married at the time of the survey. Of the 350 participants, 233(66.8%) were Muslims and 82(23.4%), 100(28.6%), and 96(27.4%) had primary, secondary, and tertiary level education, respectively. Approximately one-fifth, 72(20.6%), of the participants had no formal education. The participants were mainly formal sector employees (*n* = 132, 37.7%) and self-employed (*n* = 125, 35.7%) with 62 (17.7%) being students at the time of the study.

**Table 2:** Background characteristics of participants

Characteristics	Number	Percent
Age (years)		
< 20	31	8.86
20–35	246	70.29
36–50	62	17.71
> 50	11	3.14
Mean (SD)	29.22(9.11)	
Sex		
Male	246	73.14
Female	94	26.86
Marital status		
Never married	142	40.57
Married	182	52.00
Divorced/separated/widowed	26	7.43
Religion		
Christianity	116	33.24
Islam	233	66.76
Education		
No formal education	72	20.57
Primary	82	23.43
Secondary	100	28.57
Tertiary	96	27.43
Employment status		
Formal sector employment	132	37.71
Self-employed	125	35.71
Unemployed	31	8.86
Student	62	17.71
Donor status		
First time	192	54.86
Repeat	158	45.14

### Donor status and background characteristics

The association between donor status and background characteristics of the participants is presented in Table 3. Of the donors in the age range of 20–35 years, 137(55.7%) were first-time donors and 109(44.3%) were repeat donors. Nearly two-thirds, 7(63.6%), of donors older than 50 years were repeat donors while the remaining 4(36.4%) were first-time donors. Male donors constituted the highest number of first-time donors (*n* = 121, 63.0%), and repeat donors (*n* = 135, 85.4%). Of the 94(26.9%) females in this study, 71(36.9%) were first-time donors while 23(14.6%) were repeat donors. Further, the chi-square test in Table 3 shows a significant association (x^2^ = 11.2740, *p* = 0.004) between donor status and marital status. Almost two-thirds, 88(61.9%), of the participants who were never married were first-time donors whereas married participants accounted for the highest number (*n* = 97, 53.3%) of repeat donors. Most, 61(63.5%), of the donors with tertiary level education were first-time donors. Participants with no formal education were largely (*n* = 40, 55.6%) repeat donors. The majority of formal sector employees were repeat donors. Nearly three-fourths of donors who were unemployed (*n* = 22, 70.9%) and students (*n* = 45, 72.6%) were first-time donors. The results demonstrate a significant association (x^2^ = 15.4324, *p* = 0.001) between donor status and employment status of participants in this study.

**Table 3:** Association of donor status and socio-demographic characteristics

Characteristics	Donor Status		X^2^	*P* value
	First time	Repeat		
Age (years)				
< 20	22(70.97)	9(29.03)		
20–35	137(55.69)	109(44.31)	6.4731	0.092^a^
36–50	29(46.77)	33(53.23)		
> 50	4(36.36)	7(63.64)		
Sex				
Male	121(63.02)	135(85.44)	22.1827	< 0.001
Female	71(36.98)	23(14.56)		
Marital status				
Never married	88(61.97)	54(38.03)		
Married	85(46.70)	97(53.30)	11.2740	0.004
Divorce/separated/widowed	19(73.08)	7(26.92)		
Religion				
Christianity	72(62.07)	44(37.93)	3.7794	0.052
Islam	119(51.07)	114(48.93)		
Education				
No formal education	32(44.44)	40(55.56)		
Primary	50(60.98)	32(39.02)	8.7010	0.034
Secondary	49(49.00)	51(51.00)		
Tertiary	61(63.54)	35(36.46)		
Employment status				
Formal sector employment	62(46.97)	70(53.03)		
Self-employed	63(50.40)	62(49.60)	15.4324	0.001
Unemployed	22(70.97)	9(29.03)		
Student	45(72.58)	17(27.42)		

^a^Fisher’s exact test

### Motivators of blood donation

Table 4 presents a descriptive summary of the six motivators of blood donation participants responded to in this study. Nearly all the donors, 316(90.3%), indicated they were motivated to donate when someone they know is in need of blood. Over four-fifths of the donors endorsed good attitude of staff (*n* = 291, 83.4%) and the desire to help other people in need of blood (*n* = 298, 85.1%) as motivators. The offer of compensation (incentives) for blood donation was the least, 187(53.4%), motivator reported. Two hundred and forty-three (69.4%) and 263(75.1%) donors endorsed appeals on radio, television or from a famous person and reminder to donate when there is a shortage of blood as motivators, respectively.

**Table 4:** Motivators of blood donation

Motivator variables	Number (%)
When someone I know is in need	316(90.29)
Good attitude of staff	291(83.38)
Incentives for donation	187(53.43)
Appeals on radio, television, or from a famous person	243(69.43)
Reminder to donate when there is a shortage	263(75.14)
To help a person in need	298(85.14)

### Motivators of blood donation in first-time and repeat donors

Analysis of the association between donor status and motivational factors is presented in Table 5. One hundred and seventy-one (89.1%) of the donors who were motivated to donate when someone they know is in need were first-time donors and 145(91.8%) were repeat donors. The attitude of staff was important to the majority of both first-time donors (*n* = 161, 83.9%) and repeat donors (*n* = 130, 82.8%). Further, the desire to help a person in need of blood as a motivator was significantly associated (× 2 = 5.0953, *p* = 0.024) with donor status as approximately four-fifths of first-time donors (*n* = 156, 81.3%) and repeat donors (*n* = 142, 89.9%) endorsed this motivator. A statistically significant (x^2^ = 14.0660, *p* = < 0.001) number of the donors who endorsed incentives as a motivator were first-time donors (*n* = 120, 62.5%). Only 67(42.4%) of them were repeat donors. Slightly over three-fourths, 122(77.2%), of the participants who considered a reminder to donate when there is a shortage of blood as a motivator were repeat donors. Of the donors that endorsed appeals on radio, television or from a famous person as a motivator, 133(69.3%) were first-time donors while 110(69.6%) of them were repeat donors.

**Table 5:** Association of donor status and motivators of blood donation

Motivator variables	Donation Status		X^2^	*P* value
	First-time	Repeat		
When someone I know is in need				
Yes	171(89.06)	145(91.77)	0.7256	0.394
No	21(10.94)	13(8.23)		
Good attitude of staff				
Yes	161(83.85)	130(82.80)	0.0689	0.793
No	31(16.15)	27(17.20)		
Incentives for donation				
Yes	120(62.50)	67(42.41)	14.0660	< 0.001
No	72(37.50)	91(57.59)		
Appeals on radio, television, or from a famous person				
Yes	133(69.27)	110(69.62)	0.0050	0.944
No	59(30.73)	48(30.38)		
Reminder to donate when there is a shortage				
Yes	141(73.44)	122(77.22)	0.6622	0.416
No	51(26.56)	36(22.78)		
To help a person in need				
Yes	156(81.25)	142(89.87)	5.0953	0.024
No	36(18.75)	16(10.13)		

### Barriers to blood donation

The barriers of first-time and repeat donors were evaluated with nine barriers to blood donation and presented in Table 6. Approximately two-thirds, 223(63.7%), of the donors endorsed poor attitude of staff as a barrier to blood donation. More than half of the donors considered the level of privacy provided during pre-donation screening (*n* = 191, 54.6%) and the concern that donated blood may be sold (*n* = 178, 50.9%) as barriers. Only one-third of the donors endorsed fear of weakness after donation (*n* = 125, 35.7%), fear of needles/pain (*n* = 119, 34.1%), and inconvenience of donors clinic (*n* = 127, 36.3%) as barriers. Further, only 39.7% and 38.6% of the donors recognised fear of contagion and absence of a gift or reward as barriers to blood donation, respectively.

**Table 6:** Barriers to blood donation

Barriers	Number (%)
Poor attitude of staff	223(63.71)
Level of privacy during screening	191(54.57)
Fear of weakness after donation	125(35.71)
Fear of needles or pain	119(34.10)
Fear of contagion	139(39.71)
Fear of feeling faint or dizzy	138(39.43)
Inconvenience of donors’ clinic	127(36.29)
Donated blood may be sold	178(50.86)
Absence of a gift or reward	133(38.55)

### Barriers to blood donation in first-time and repeat donors

Result of the association of donor status and deterrents to blood donation is presented in Table 7. Poor attitude of staff at the donors’ clinic was a major barrier among first-time donors 120(62.5%) and repeat donors 103(65.2%). Privacy during pre-donation screening was an important barrier for more than half of both first-time donors 109(56.8%) and repeat donors 82(51.9%). Of the number that was deterred by the concern that donated may be sold, 112(58.3%) were first-time donors while 66(41.8%) were repeat donors. As illustrated in Table 7, more of repeat donors than first-time donors indicated they were not deterred by weakness after donation (74.1 vs 56.3% *p* = 0.001), fear of needles/pain (76.6 vs 57.1%, *p* = < 0.001), or inconvenience of donors clinic (72.2 vs 56.8%, *p* = 0.003). The results also show that only 86(44.8%) of first-time donors and 53(33.5%) of repeat donors considered fear of contagion as a barrier to blood donation (x^2^ = 4.5796, *p* = 0.032). Most of the donors, 109(57.4%) of first-time donors and 103(66.5%) of repeat donors, did not consider the absence of gift/reward (incentives) as a barrier to blood donation.

**Table 7:** Association of donor status and barriers to blood donation

Barriers	Donor Status		X^2^	*P* value
	First-time	Repeat		
Poor attitude of staff				
Yes	120(62.50)	103(65.19)	0.2713	0.602
No	72(37.50)	55(34.81)		
Level of privacy during screening				
Yes	109(56.77)	82(51.90)	0.8299	0.362
No	83(43.23)	76(48.10)		
Fear of weakness after donation				
Yes	84(43.75)	41(25.95)	11.9620	0.001
No	108(56.25)	117(74.05)		
Fear of needles or pain				
Yes	82(42.93)	37(23.42)	14.6536	< 0.001
No	109(57.07)	121(76.58)		
Fear of contagion				
Yes	86(44.79)	53(33.54)	4.5796	0.032
No	106(55.21)	105(66.46)		
Fear of feeling faint or dizzy				
Yes	93(48.44)	45(28.48)	14.4537	< 0.001
No	99(51.56)	113(71.52)		
Inconvenient of donor clinic				
Yes	83(43.23)	44(27.85)	8.8693	0.003
No	109(56.77)	114(72.15)		
Donated blood may be sold				
Yes	112(58.33)	66(41.77)	9.5118	0.002
No	80(41.67)	92(58.23)		
Absence of a gift or reward				
Yes	81(42.63)	52(33.55)	2.9730	0.085
No	109(57.37)	103(66.45)		

### Blood donor’s knowledge of blood donation

Table 8 summarize donors’ knowledge about blood donation. Only a little over one-third of the donors knew the minimum age for blood donation (*n* = 126, 36.0%) and the maximum number of donations per year (*n* = 132, 37.7%). As illustrated in Table 8, 152(43.4%) of the participants answered correctly that the interval between two donations is 4 month. Three-fourths, 264(75.4%), of the donors knew that donated blood expires. Almost all, 319(91.4%), the participants answered correctly that a person positive for HIV/AIDS is not eligible to donate blood.

**Table 8:** Blood donor’s knowledge about blood donation

Knowledge variables	Number	Percent
Minimum age for blood donation		
< 17 years	59	16.86
17–18 years	126	36.00
> 18 years	165	47.14
Maximum number of donations in a year		
Once	81	23.14
Twice	93	26.57
Thrice	132	37.71
Four or more times	44	12.57
Interval between two blood donations		
< 4 months	136	38.86
4 months	152	43.43
5 or more months	62	17.71
Does donated blood expire		
Yes	264	75.43
No	86	24.57
HIV/AIDS affected persons can donate blood		
Yes	30	8.60
No	319	91.40

## Discussion

To meet the blood transfusion demand and ensure adequate and constant supply of blood in all hospitals where blood transfusion is performed, it is essential to understand the motivators and barriers to blood donation for the formulation and effective implementation of donor recruitment programmes. In this hospital-based survey, we found that the donors were predominantly first-time donors, which contradicts findings reported in earlier studies [14, 19]. The inconsistency may be due to the fact that the earlier studies were conducted in developed countries. One important implication of the finding is that the hospital may be unable to ensure adequate, constant, and safe blood supply since the majority of its donors are first-time donors who may not return to donate. Further, Allain argue that the risk of infection is high in first-time donors compared to repeat donors [20]. Hence, to ensure a safe and adequate blood supply, management of the donor clinic need to institute strategies to retain first-time donors as voluntary regular donors.

Evidence from a review in sub-Saharan Africa indicates that altruism is the most common motivator associated with the return of donors for more donations in the region [11]. For all donor groups in this study, the desire to help a family member or a friend in need of blood was the major motivator, which is inconsistent with studies in Germany [14], Saudi Arabia [16] and Senegal [21]. Altruistic donors, unlike those in this study, are influenced by the desire to help others and to improve the health of people they may never meet [1]. Contrary to this, the majority of donors in this study donated to meet the transfusion needs of hospitalised friends or family members and not to help others they do not know. However, it is important to note that some recipients prefer blood from a relative or family member because they belief characteristics of a donor can be transferred to the recipient through the blood. In Cameroon Koster and Hassall found that donating blood to a relative was considered much more acceptable compared with donating blood to an institution or a stranger who may be undeserving of the donor’s family ‘good’ blood [12]. This may explain why donors in the current study donated blood primarily to save the life of either a family member or a friend.

Good attitude of staff at the donor clinic, a donors desire to help other people, and a reminder to donate when there is a shortage of blood were also endorsed as strong motivators for blood donation. Shaz et al. reported similar findings among African Americans in the United State [22]. Consistent with our finding, awareness of blood shortage was reported in Senegal as one of two main motivators of blood donation [21]. In addition, when Mauka and colleagues surveyed blood donors in Tanzania, they found that good previous donation experience was significantly associated with repeat donation [23]. This suggests that friendly attitude of staff and a good relationship between staff and donors may influence the return of donors to donate more. Similar to a previous study [24], the offer of incentives was the least endorsed motivator in this study which is in contrast to a study in South Africa [12]. In the South African study incentives in the form of gift items, time off work, money, recognition among others was the second most endorsed motivator [12]. The concept of incentives for donation may be less important in this study because the participants were predominantly first-time donors donating blood for either a family member or a friend.

The current study found that poor attitude of staff was the major reported barrier to blood donation among all the donor groups. However, more repeat donors than first-time donors endorsed the factor as a barrier. Likewise, Shaz et al. reported poor attitude of staff as a deterrent when they surveyed African Americans and white blood donors [22]. This finding explains why the good attitude of staff was endorsed as one of the major motivators in this study. Donors feel satisfied and motivated to return when they receive good care. This suggests that effective communication, counselling, and friendly attitude of staff have the potential to motivate donors and eliminate barriers to blood donation. The level of privacy provided during pre-donation screening and the concern that donated blood may be sold to patients in need of blood were also endorsed as strong barriers to blood donation. Similarly, Shaz et al. reported privacy during pre-donation screening as a factor that influences the decision to donate [22]. Regarding the concern that donated blood may be sold, a study in India reported similar concerns in 4.2% of college students, which is lower than what was found in this study [25]. Privacy during pre-donation screening and the concern that donated blood may be sold were more important to first-time donors compared to repeat donors in this study.

For all donor groups, fear of weakness, fear of needles/pain, and fear of contagion were not recognised as important barriers to blood donation. Similarly, when Shaz et al. surveyed college age blood donors they found that pain, feeling of faint and dizziness were not important deterrents [15]. Likewise, Alfouzan reported fear of needles and pain in only 37.6% and 25.9% when he surveyed blood donors in Saudi Arabia, respectively [16]. However, Asamoah-akuoko and colleagues reported fears related to pain, adverse effects, and contagion as an important deterrent when they review studies on blood donation in sub-Saharan Africa [11].

The convenience of a blood donation centre, the times at which it open and waiting times can act as barriers to blood donation. Studies have shown that both first-time and repeat donors are motivated to donate when the process involves little or no disruption to their normal activities [1]. In this study, the inconvenience of donor clinic and absence of gift/reward were not considered important barriers to blood donation. Consistent with this finding, Yuan et al. reported unappealing incentives as the least rank deterrent among donors at a university campus in California. However, they reported inconvenient operating time and location of the donor centre among the top deterrents in their study, which is inconsistent with our finding [26]. Likewise, Schlumpf et al. reported the lack of a convenient place to donate as a major deterrent to blood donation [27]. Our finding may be attributed to the fixed location of the clinic, its nearness to the town centre, and the availability of reliable transport to the facility, as these factors are thought to ease inconvenience [1].

We found that more than half of the donors in the current study did not know the minimum age for blood donation, maximum number of donations in a year, and the recommended interval between two donations. The current results are consistent with the results of an earlier study in Saudi Arabia [16] but contradict those of Jemberu et al in Ethiopia [28]. These findings underscore the need for blood donation education campaigns in communities, on the radio, and television to educate the public about blood donation. However, almost all the donors in this study knew that persons positive for HIV/AIDS are not eligible to donate blood.

## Conclusion

We found that donors desire to help a family member or a friend in need of blood was the most cited motivator for blood donation in this study followed by a positive attitude of staff at the donor clinic, the desire to help other people, and a reminder to donate when there is a shortage of blood. Poor attitude of staff was reported as the major barrier to blood donation followed by the level of privacy provided during pre-donation screening and the concern that donated blood may be sold to patients in need of blood. Our findings suggest that public education on blood donation, regular prompts of donors to donate when there is a shortage, and friendly attitude of staff have the potential to motivate donors and eliminate barriers to blood donation.

## Abbreviations

HIV/AIDS: Human Immunodeficiency Virus/Acquired Immunodeficiency Syndrome
TTH: Tamale Teaching Hospital
WHO: World Health Organisation
